# Curative procedures of oral health and structural characteristics of primary dental care

**DOI:** 10.11606/S1518-8787.2018052016291

**Published:** 2018-03-14

**Authors:** Alexandre Baumgarten, Fernando Neves Hugo, Alexandre Fávero Bulgarelli, Juliana Balbinot Hilgert

**Affiliations:** I Universidade Federal do Rio Grande do Sul. Programa de Pós-Graduação em Epidemiologia. Porto Alegre, RS, Brasil; II Universidade Federal do Rio Grande do Sul. Programa de Pós-Graduação em Saúde Coletiva. Porto Alegre, RS, Brasil; III Universidade Federal do Rio Grande do Sul. Programa de Pós-Graduação em Odontologia. Porto Alegre, RS, Brasil

**Keywords:** Dental Health Services, supply & distribution, Public Health Dentistry, Comprehensive Dental Care, Primary Health Care, Health Care Quality, Access, and Evaluation, Oral Health

## Abstract

**OBJECTIVE:**

To evaluate if the provision of clinical dental care, by means of the main curative procedures recommended in Primary Health Care, is associated with team structural characteristics, considering the presence of a minimum set of equipment, instrument, and supplies in Brazil’s primary health care services.

**METHODS:**

A cross-sectional exploratory study based on data collected from 18,114 primary healthcare services with dental health teams in Brazil, in 2014. The outcome was created from the confirmation of five clinical procedures performed by the dentist, accounting for the presence of minimum equipment, instrument, and supplies to carry them out. Covariables were related to structural characteristics. Poisson regression with robust variance was used to obtain crude and adjusted prevalence ratios, with 95% confidence intervals.

**RESULTS:**

A total of 1,190 (6.5%) dental health teams did not present the minimum equipment to provide clinical dental care and only 2,498 (14.8%) had all the instrument and supplies needed and provided the five curative procedures assessed. There was a positive association between the outcome and the composition of dental health teams, higher workload, performing analysis of health condition, and monitoring of oral health indicators. Additionally, the dental health teams that planned and programmed oral health actions with the primary care team monthly provided the procedures more frequently. Dentists with better employment status, career plans, graduation in public health or those who underwent permanent education activities provided the procedures more frequently.

**CONCLUSIONS:**

A relevant number of Primary Health Care services did not have the infrastructure to provide clinical dental care. However, better results were found in dental health teams with oral health technicians, with higher workload and that plan their activities, as well as in those that employed dentists with better working relationships, who had dentists with degrees in public health and who underwent permanent education activities.

## INTRODUCTION

Comprehensiveness is considered a priority among the doctrinal principles of the Brazilian Unified Health System (acronym in Portuguese is SUS), since it means taking care of all users’ needs. That goes from ensuring actions and services to have their conditions taken care of and to providing access to all healthcare technologies. Comprehensiveness of assistance begins, traditionally, in primary care, which is the first point of contact of the people with the health system, where they receive care for most of their everyday health needs. In dentistry, primary care includes health promotion, disease prevention, and diagnosis and treatment of acute and chronic dental diseases in a variety of clinical care settings. Thus, the services are offered respecting users’ demands and the availability of technologies^6^.

The improvement of healthcare depends of modifications in the process or structure, since the results are always a consequence of a given change^6^. Thus, the capacity of a health system to provide effective services is influenced directly by infrastructure conditions and the availability and adequacy of equipment, materials, and supplies that meet the real needs of the service^21^. For dentistry, invariably, the possibility of providing good quality care depends deeply on the availability of such, which have a significant impact on the local health system^14^. Problems in this organizational sphere compromise the assistance, the quality of the service provided and the achievement of work goals, precluding the execution of clinical procedures effectively, making it necessary to refer users to others services^17^. These problems are also seen as contributing factors for the abandonment of dental care and for limiting the access to oral health services^13^.

The Brazilian model of health care, which has Primary Health Care (PHC) as its main point of entry, aims to care for the health problems of high prevalence and relevance. Thus, the dental care established and provided in PHC deals with the most prevalent oral diseases. With the inclusion of the dental health team (DHT) in the Family Health Strategy, it became possible to establish an effective dental practice. This may be further favored by the inclusion of an oral health technician, for example, which may eventually produce a positive impact in work processes, or by the establishment of a workload of at least two shifts per day, which is essential for improving access to care. Likewise, permanent education is a professional qualification strategy that allows the creation of a space of practices for the reorientation of work processes that may ultimately lead to an improvement in the quality of care.

The importance of structure evaluation in primary care dentistry is evident, as it is essential for identification and characterization of fragilities in PHC, from the perspective of efficiency and improvement of quality and access to oral health care. A single regional study that evaluated the relationship between structure and primary dental care provision was identified^9^, and there are no previous studies evaluating which structural factors are relevant for a better clinical performance of DHT within PHC.

Thus, in the scope of an evaluative study of PHC DHT, this study may contribute in mapping the provision of a set of clinical dental care procedures (e.g. curative actions), in addition to providing further analysis about the public oral health network in Brazil. Through a national analysis of the structure of DHT within PHC, this study seeks to support the decision-making process, reflecting in the construction of subsidies to guide management processes, as well as to strengthen oral health actions.

Given that, the care provided is directly related to the clinical performance of the DHT and to structural characteristics of the PHC services. The aim of this study is to evaluate if the provision of clinical dental care, by means of the main curative procedures recommended in PHC, is associated with team’s structural characteristics, considering the presence of a minimum set of equipment, instrument, and supplies in Brazil’s primary health care services.

## METHODS

A cross-sectional exploratory study with multicenter data collection across Brazil, carried out between March and December of 2014, in 24,055 Primary Health Care Teams that adhered to the second cycle of the National Program for Access and Quality Improvement (PMAQ). For this study, 18,114 PHC with DHT were assessed, corresponding to 81.5% of the DHT in Brazil.

For data collection, external evaluators were selected and uniformly trained with a field manual. The data collection was carried out *in loco* using tablets, each containing an app with a previously tested and standardized instrument. A data collection qualification protocol was established and consisted of five criteria: correspondence of geographical coordinates captured by the instrument, time length of the data collection per module, start and end hours of the assessment, ratio of unanswered questions and answers with repeated characters. The questionnaire was applied during an interview, in which info was gathered by means of provision of documentation on identification, functioning, and structure of PHC services and assessment, *in loco*, of dental equipment, instruments and dental supplies. In addition, data were collected by interviewing a member of the dental health team.

The DHT eligible for the study were those with dental equipment to conduct curative/restorative dental procedures in the facility^4^. The dental equipment were assessed by the presence of the following variables: dental chair that went up and down and tilted, dental cart, light reflector, basin, saliva ejector machine, high and low speed handpieces, air compressor with safety valve and dental stool (exclusion in Figure).

**Figure:**
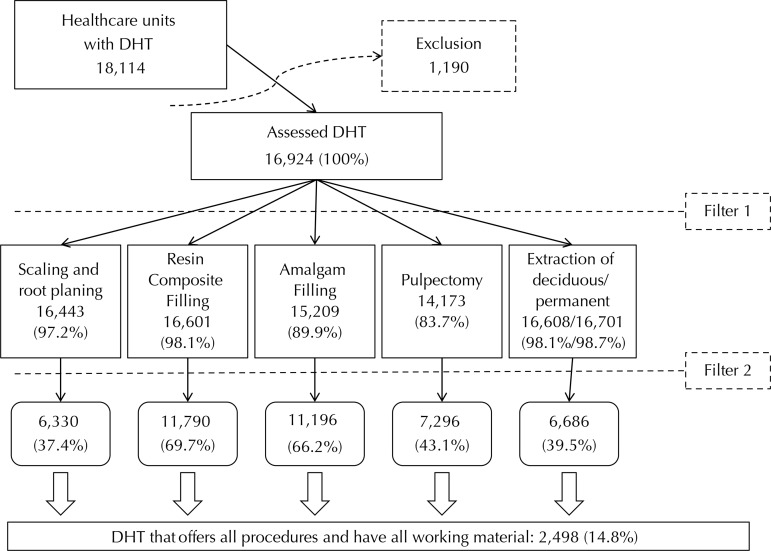
Description of the outcome, considering curative procedures and materials available.

DHT: dental health teams

Exclusion: lack of minimum dental equipment for service.

Filter 1: procedure carried out in the healthcare center.

Filter 2: minimum material available to carry out the procedure.

The assessment of the procedures was made by asking the dentist: “Does the dental health team perform the following procedures?”. 1) Scaling and root planning; 2) Resin composite filling; 3) Amalgam filling; 4) Pulpectomy and; 5) Simple tooth extraction (deciduous and permanent teeth).

The structure for the execution of the procedures was assessed according to equipment, instruments, and supplies available. For all procedures assessed: mouth mirror, explorer, cotton tweezers, personal protective equipment (glove, protective eyewear, surgical mask, protective clothing and hood), saliva ejector and anesthetics material (anesthetic and carpule syringe with needle) was mandatory, with exception of the latter for scaling and root planning. Besides these, the equipment, instruments, and dental supplies required for each procedure are presented next: 1) Scaling, root planning: periodontal probes, pads, ultrasound or curettes with sharpening stone; 2) Resin composite filling: photopolymerizer, calcium hydroxide and its applicators, dental drills, excavators for dentin, composite resin, acid and adhesive system, insertion spatulas, dental microbrush, articulating paper, cotton rolls; 3) Amalgam filling: calcium hydroxide and its applicators, amalgam burnishers and condensers, dental drills, excavators for dentin, amalgam carrier, articulating paper, amalgam capsule with amalgamator or amalgam set for manual preparation; 4) Pulpectomy: dental drills, excavators for dentin, endodontic files or barbed broach, irrigation syringes, intracanal medications, temporary restorative materials, cotton rolls; 5) Simple tooth extraction: bone forceps, scalpel handle and blade, elevators, surgical curettes, child and adult forceps, bone files, needle carrier, syndesmotome, surgical scissor, dental suture thread, pads, disposable syringe for irrigation.

The exploratory variables assessed and related to the structure were the following: i) dental health team modality (modality I, including one doctor of dental surger and one oral health assistant (OHA); modality II, including one doctor of dental surger, one DHA and one oral health technician; and parameterized DHT including two doctor of dental surger (working 20 hours per week/each), one OHA and one OH technician); ii) quantity of shifts and days in service, per week; iii) conduction of a health status analysis (yes or no); iv) monitoring and analysis of the dental health indicators and planning of activities and scheduling of actions (yes or no). The exploratory variables of the dentist were: (i) employment status (Temporary Agreement; Statutory Public Employee; Commissioned Role; Self-employed or other; Public employee under Brazilian Employment Law (CLT); CLT agreement); (ii) career plan (yes or no); (iii) graduate studies in public health or family health (residency program, specialization degree, master’s degree or doctor’s degree); (iv) permanent education (yes or no).

The outcome was created from the confirmation of five curative procedures performed by the doctor of dental surger in PHC, considering the presence of minimum equipment, instrument and supplies to the execution of these. The outcome was dichotomized in: 1) yes when the dentist carried out the procedure and had all the materials, and 2) no.

Data were analyzed using the software SPSS v21 (SPSS Inc.; Chicago). The chi-square test was used to analyze the existence of associations between the independent variables and the provision of curative actions. Poisson regressions with robust variance were used to obtain the crude and adjusted prevalence ratios with their respective 95% confidence intervals. In the adjusted model, we included theoretically relevant variables, which show some degree of association during the analyses. Stratified analyses by procedures were also performed. The project was approved by Universidade Federal do Rio Grande do Sul’ Ethics Committee under Process 21904.

## RESULTS

This study assessed 18,114 dental health teams all over Brazil. Of those, 1,190 were not analyzed because the dental equipment needed to carry out the curative or restorative procedures was not available in the PHC service (Exclusion, as presented in Figure). The 16,924 remaining DHT were assessed in this study and the frequencies of procedures are shown in Figure. Resin composite fillings (16,601), permanent (16,608) and deciduous (16,701) teeth extractions were performed more frequently by DHT in the PHC, while 14,173 performed pulpectomy (after filter one, Figure).

The number of DHT with minimal structure to the provision of the procedures with all materials was, as follows: 6,330 for scaling and root planning, 11,790 for resin composite filling, 11,196 for amalgam filling, 7,296 for pulpectomy, and 6,686 for simple tooth extraction. Only 2,498 DHT (14.8%) had all assessed items (after the second filter application [Material Filter], Figure). Table 1 describes the presence or lack of equipment, instruments, and dental supplies in the remaining 14,426 DHT, depicting an overview of Brazil’s primary care dentistry structure.

**Table 1:** Description of equipment, instruments and dental supplies in the PHC services with a negative outcome. (n = 14,426)

Variable	With material	Without material

	n (%)	n (%)
Equipment		
Amalgamator	12,683 (87.9)	1,743 (12.1)
Photopolymerizer	13,973 (96.9)	453 (3.1)
Saliva ejector	14,279 (98.9)	147 (1.1)
Dental ultrasound	3,111 (21.9)	11,315 (78.1)
Instrument		
Elevators	14,267 (98.9)	159 (1.1)
Bone forceps	11,531 (79.9)	2,895 (20.1)
Calcium hydroxide applicators	13,877 (96.2)	549 (3.8)
Amalgam burnishers	13,609 (94.3)	817 (5.7)
Scalpel handle	13,018 (90.2)	1,408 (9.8)
Endodontic aspiration cannula	2,227 (15.7)	12,199 (84.3)
Amalgam condensers	13,236 (91.8)	1,190 (8.3)
Surgical curettes	12,429 (86.2)	1,997 (13.8)
Periodontal curettes	13,263 (91.9)	1,163 (8.6)
Dentin excavators	13,746 (95.3)	680 (4.7)
Carvers	12,993 (90.1)	1,433 (9.9)
Resin insertion spatulas	13,156 (91.2)	1,270 (8.8)
Endodontic files or barbed broach	7,793 (54.0)	6,633 (46.0)
Child and adult forceps	14,252 (98.8)	174 (1.2)
Bone files	9,243 (64.1)	5,183 (35.9)
Periodontal curette with sharpening stones	6,070 (42.1)	8,356 (57.9)
Needle carrier	14,018 (97.2)	408 (2.8)
Amalgam carrier	13,161 (91.2)	1,265 (8.8)
Carpule syringes	14,298 (99.1)	128 (0.9)
Syndesmotomes	12,794 (88.7)	1,632 (11.3)
Millimetric probes	6,082 (42.2)	8,344 (57.8)
2Surgical ejectors	3,460 (24.0)	10,966 (76.0)
Surgical scissor	13,466 (93.4)	960 (6.6)
Dental tools (set of three)^a^	13,777 (95.5)	649 (4.5)
Supplies		
Acid and adhesive system	14,008 (97.1)	418 (2.9)
Amalgam (capsule)	9,043 (62.7)	5,383 (37.3)
Amalgam (manual preparation)	1,889 (13.1)	12,537 (86.9)
Anesthetic	14,211 (98.5)	215 (1.5)
Various drills	14,210 (98.5)	216 (1.5)
PPE^b^	14,168 (98.2)	258 (1.8)
Dental suture thread	14,085 (97.6)	341 (2.4)
Pads	14,174 (98.2)	252 (1.8)
Scalpel blades	13,164 (91.2)	1,262 (8.8)
Temporary filling material	13,962 (96.8)	464 (3.2)
Intracanal medications	10,290 (71.3)	4,136 (28.7)
Dental microbrushes	13,359 (92.6)	1,067 (7.4)
Articulating papers (carbon paper)	13,083 (90.7)	1,343 (9.3)
Photopolymerizing resins	14,150 (98.1)	276 (1.9)
Cotton rolls	14,020 (97.2)	406 (2.8)
Disposable syringes for irrigation	10,638 (73.7)	3,788 (26.3)

^a^ Mouth mirror, explorer, and cotton tweezers.

^b^ Personal Protective Equipment: Glove, protective eyewear, surgical mask, protective clothing, and hood.

Results of the outcome and its association with exploratory variables are presented in Table 2. The outcome was significantly more frequent in teams of modality II and parameterized; that were open in three shifts, and six days of the week; DHT that performed analysis of the health status; monitored and analyzed oral health indicators; that programmed and planned activities; and whose dentist was a public employee under Brazilian Employment law; had career plan; had graduate studies in public or family health and that took part of Permanent Education.

**Table 2:** Distribution between exploratory variables assessed in respect to the provision of curative procedures of oral health.

Variable	Provision of curative dental procedures		

	Yes	No	p*

	n (%)	n (%)	
Dental Health Team Modality			> 0.001
Dental Health Team – Modality I	1,852 (12.9)	12,495 (87.1)	
Dental Health Team – Modality II	588 (25.8)	1,691 (74.2)	
Dental Health Team – Parameterized	58 (19.5)	240 (80.5)	
Service shifts			> 0.001
1 shift	89 (7.0)	1,190 (93.0)	
2 shifts	2,165 (14.5)	12,763 (85.5)	
3 shifts	244 (34.0)	473 (66.0)	
Days of operation			0.004
1–4 days	2,374 (15.0)	13,458 (85.0)	
5 days	118 (11.2)	933 (88.8)	
6 days	6 (14.6)	35 (85.4)	
Analysis of the health status			> 0.001
No	328 (7.5)	4,073 (92.5)	
Yes	2,170 (17.3)	10,353 (82.7)	
Monitoring and analysis of the oral health indicators			> 0.001
No	460 (8.2)	5,123 (91.8)	
Yes	2,038 (18.0)	9,303 (82.0)	
Action programming and planning activity			> 0.001
No	342 (11.9)	2,521 (88.1)	
Yes, along with the primary care team	385 (11.7)	2,900 (88.3)	
Yes, only with the dental health team	1,771 (16.4)	9,005 (83.6)	
Employment Relationship			> 0.001
Temporary agreement	627 (10.5)	5,367 (89.5)	
Statutory public employee	1,174 (15.8)	6,274 (84.2)	
Commissioned role	49 (15.4)	270 (84.6)	
Self-employed or other	20 (9.1)	199 (90.9)	
Public employee (under CLT)	267 (24.7)	813 (75.3)	
CLT agreement	356 (20.7)	1,364 (79.3)	
Career plan			> 0.001
No	1,672 (12.8)	11,399 (87.2)	
Yes	782 (23.2)	2,584 (76.8)	
Graduate studies in Public or Family Health			> 0.001
No	1,504 (12.4)	10,644 (87.6)	
Yes	994 (20.8)	3,782 (79.2)	
Permanent education			> 0.001
No	162 (5.2)	2,965 (94.8)	
Yes	2,336 (16.9)	11,461 (83.1)	

CLT: Brazilian Employment Law

* Chi-square test.

The unadjusted analysis was somewhat similar to the adjusted, with exception of number of workdays per week (Table 3). When the variables regarding the DHT modalities were assessed, Modality II (PR = 1.16, 95%CI 1.14–1.19) and parameterized DHT (PR = 1.11, 95%CI 1.03–1.19) had better performance. Regarding the working hours, DHT that opened two (PR = 1.34, 95%CI 1.27–1.42) and three (PR = 1.63, 95%CI 1.54–1.74) shifts per day, and DHT with five (PR = 1.13, 95%CI 1.07–1.19) and six (PR = 1.25, 95%CI 1.04–1.50) days of operation per week also had better performance. DHT in which management provided information to aid in the health status assessment (PR = 1.14, 95%CI 1.11–1.17) and that monitored and analyzed dental health indicators (PR = 1.12, 95%CI 1.10–1.15) had better performance. Additionally, DHT that planned and scheduled its actions monthly within PHC also performed better (PR = 1.03, 95%CI 1.01–1.06).

**Table 3:** Crude and adjusted analysis between the outcome and associated factors.

Variables	Unadjusted analysis		Adjusted analysis	
	
	PR	95%CI	PR	95%CI
Dental Health Team Modality				
Dental Health Team – Modality I	1	-	1	-
Dental Health Team – Modality II	1.29	1.26–1.32	1.16	1.14–1.19
Dental Health Team – Parameterized	1.10	1.03–1.18	1.11	1.03–1.19
Service shifts				
1 shift	1	-	1	-
2 shifts	1.34	1.28–1.40	1.34	1.27–1.42
3 shifts	1.89	1.80–1.99	1.63	1.54–1.74
Days of operation				
1–4 days	1	-	1	-
5 days	0.90	0.86–0.93	1.13	1.07–1.19
6 days	1.01	0.83–1.22	1.25	1.04–1.50
Analysis of the health status				
No	1	-	1	-
Yes	1.32	1.28–1.35	1.14	1.11–1.17
Monitoring and analysis of the oral health indicators				
No	1	-	1	-
Yes	1.28	1.26–1.31	1.12	1.10–1.15
Action programming and planning activity				
No	1	-	1	-
Yes, along with the primary care team	1.05	1.01–1.08	0.98	0.95–1.01
Yes, only with the dental health team	1.15	1.12–1.19	1.03	1.01–1.06
Employment Relationship				
Temporary agreement	1	-	1	-
Statutory public employee	1.16	1.14–1.19	1.09	1.06–1.12
Commissioned role	1.08	1.00–1.16	1.05	0.98–1.13
Self-employed or other	0.98	0.90–1.08	0.99	0.91–1.08
Public employee (under CLT)	1.36	1.32–1.41	1.27	1.23–1.32
CLT agreement	1.30	1.26–1.34	1.16	1.13–1.20
Career plan				
No	1	-	1	-
Yes	1.22	1.19–1.24	1.10	1.08–1.13
Graduate studies in public or Family Health				
No	1	-	1	-
Yes	1.18	1.16–1.20	1.07	1.05–1.09
Permanent education				
No	1	-	1	-
Yes	1.41	1.37–1.45	1.23	1.19–1.27

CLT: Brazilian Employment Law

The type of employment contract was also critical for the outcome; the best results were achieved by statutory employees (PR = 1.09, 95%CI 1.06–1.12), as well as by public employees (PR = 1.27, 95%CI 1.23–1.32) and by those whose contract was managed by the Brazilian Employment Law (CLT – *Consolidação das Leis do Trabalho*) (PR = 1.16, 95%CI 1.13–1.20). Additionally, dentists with career plans (PR = 1.10, 95%CI 1.08–1.13), graduate studies in public or family health (PR = 1.07, 95%CI 1.05–1.09) or those who underwent permanent education activities (PR = 1.23, 95%CI 1.19–1.27) were more likely to provide all procedures with the required material (Table 3).

Table 4 presents adjusted prevalence ratios stratified by each procedure. The results were similar and followed the same direction as those found and described in Table 2. It is worth mentioning that simple tooth extractions had no association with any of the studied exploratory variables, except for a third shift (PR = 1.01, 95%CI 1.01–1.02) and permanent education (PR = 1.02, 95%CI 1.00–1.03), even though they were of small magnitude.

**Table 4:** Multivariate Analyses between each procedure and associated factors.

Variable		Scaling and root planning		Resin		Amalgam		Pulpectomy		Simple tooth extraction	
				
		PR	95%CI	PR	95%CI	PR	95%CI	PR	95%CI	PR	95%CI
Dental Health Team Modality	Dental Health Team – Modality I	1	-	1	-	1	-	1	-	1	-
	Dental Health Team – Modality II	1.34	1.28–1.40	1.02	0.99–1.05	1.03	1.00–1.06	1.29	1.24–1.34	1.00	0.99–1.01
	Dental Health Team – Parameterized	1.30	1.15–1.47	0.99	0.92–1.08	1.01	0.92–1.10	1.23	1.10–1.38	0.98	0.96–1.00
Service shifts	1 shift	1	-	1	-	1	-	1	-	1	-
	2 shifts	1.65	1.45–1.87	1.15	1.09–1.22	1.24	1.16–1.33	1.74	1.56–1.94	1.01	0.99–1.02
	3 shifts	2.44	2.14–2.80	1.22	1.13–1.31	1.30	1.20–1.41	2.35	2.09–2.64	1.02	1.00–1.03
Days of operation	1–4 days	1	-	1	-	1	-	1	-	1	-
	5 days	1.07	0.97–1.20	1.11	1.04–1.17	1.09	1.02–1.16	1.19	1.09–1.30	1.00	0.99–1.02
	6 days	1.55	1.10–2.20	0.78	0.57–1.07	0.90	0.66–1.22	2.26	1.80–2.84	1.00	0.95–1.05
Analysis of the health status	No	1	-	1	-	1	-	1	-	1	-
	Yes	1.20	1.13–1.27	1.10	1.07–1.13	1.11	1.07–1.14	1.18	1.13–1.13	1.00	0.99–1.01
Monitoring and analysis of the Oral Health Indicators	No	1	-	1	-	1	-	1	-	1	-
	Yes	1.22	1.15–1.28	1.06	1.03–1.09	1.11	1.08–1.14	1.12	1.08–1.17	1.00	0.99–1.01
Action programming and planning activity	No	1	-	1	-	1	-	1	-	1	-
	Yes, along with the basic health team	1.06	1.00–1.13	1.04	1.01–1.07	1.07	1.04–1.11	0.96	0.92–1.01	1.01	0.99–1.01
	Yes, only with the dental health team	1.00	0.93–1.07	1.01	0.97–1.05	1.00	0.96–1.04	0.92	0.86–0.97	1.00	0.99–1.01
Employment relationship	Temporary agreement	1	-	1	-	1	-	1	-	1	-
	Statutory public employee	1.01	1.04–1.16	1.02	0.99–1.04	1.10	1.07–1.13	1.17	1.11–1.22	0.99	0.99–1.00
	Commissioned role	1.08	0.93–1.25	1.00	0.93–1.08	1.04	0.96–1.13	1.12	0.99–1.28	1.01	0.99–1.02
	Self-employed or other	0.92	0.75–1.14	1.03	0.94–1.12	0.96	0.86–1.07	1.09	0.93–1.29	0.99	0.98–1.02
	Public employee (under CLT)	1.37	1.27–1.47	1.15	1.11–1.20	1.16	1.12–1.21	1.50	1.41–1.60	0.99	0.99–1.09
	CLT agreement	1.25	1.17–1.33	1.02	0.98–1.04	1.10	1.06–1.15	1.46	1.39–1.54	1.00	0.99–1.01
Career plan	No	1	-	1	-	1	-	1	-	1	-
	Yes	1.28	1.22–1.34	1.00	0.97–1.03	0.91	0.96–1.02	1.22	1.18–1.27	1.00	0.99–1.01
Public health graduation	No	1	-	1	-	1	-	1	-	1	-
	Yes	1.19	1.14–1.24	0.99	0.97–1.02	1.03	1.00–1.05	1.11	1.08–1.15	1.00	0.99–1.01
Permanent education	No	1	-	1	-	1	-	1	-	1	-
	Yes	1.36	1.27–1.46	1.10	1.06–1.13	1.14	1.10–1.18	1.44	1.35–1.53	1.01	1.01–1.02

CLT: Brazilian Employment Law

## DISCUSSION

The study points important results for Brazil’s dental public health, indicating that only 14.8% of the 18,114 Brazilian DHT can offer a minimum set of clinical procedures, considering the presence of a minimum set of equipment, instruments and dental supplies. This emphasizes the need for improvement in the existing structure to ensure dental care of quality in PHC. This is probably because the DHT do not have access to a minimum set of equipment, instruments, and supplies that are essential for the provision of five simple curative procedures that should be provided in any PHC service. The National Policy on Oral Health significantly increased the access to primary dental care and to dental specialties making efforts to embrace oral health care within an oral health network in all Brazilian territory^1^ ^,^ ^4^. However, despite the consolidated funding of this policy, it does not seem to be enough to guarantee the provision of a minimum set of dental curative actions^10^.

Decentralization gave municipalities the role of organizing and providing primary health care and, as such, purchasing and maintaining supplies and equipment. This requires managerial competence, with technical and administrative capacity to ensure the availability of equipment, materials, and supplies that are essential in PHC services. However, the fact that 1,190 DHT lack minimum work infrastructure, as well as the fact that several curative procedures of lower complexity are not carried out – those that comprise most of the population’s healthcare needs – is a challenge to the effectiveness of PHC. Even though several positive results have been obtained, universalization still excludes many people and there is selective comprehensiveness of dental health care^3^ ^,^ ^12^.

The results demonstrated that some factors might be critical to ensure assistance in dental primary healthcare. The DHT that had oral health technician – a professional that performs tasks assigned for direct patient care were able to streamline dental work, increase productivity, quality and contribute to positive changes in PHC practices that allow the dentist to focus on more complex activities – emphases the importance of teamwork in PHC which may ultimately lead to comprehensiveness^20^. Thus, teamwork contributes to a professional practice that rebuilds itself in the other’s practice and is made easier by adding up feedback from all professionals that comprise the team, with greater impact on the different factors that interfere with the health/disease process and in the organization of the work structure^18^. Tooth extraction occurs independently of factors involving work characteristics, monitoring of indicators as well as healthcare models or questions involving the dentists. It has been historically performed in Brazil’s Dental Public Health services and recent evidence supports that it does not correlate to human development index, to the vulnerability of the population or to service location^5^. This may explain the associations of small magnitude with only two of the studied variables.

Permanent education and teamwork are considered critical aspects of the quality of PHC. It may promote professional competence, team integration and facilitate interdisciplinarity^16^. The provision of dental care of quality is dependent on the presence of equipment, instruments, and supplies of good quality an in sufficient quantity. The physical structure of the working environment directly influences the productivity of the dentists^1^ ^,^ ^19^. Brazil needs to improve the working conditions, as well as the infrastructure needed by DHT^15^ as this may ultimately lead to improved access and comprehensiveness of public dental care, two of the main guidelines of Brazil’s National Oral Health Policy^1^ ^,^ ^7^.

The planning and scheduling of monthly actions, with all the primary care team, has given a new dimension to Brazil’s PHC, in the sense of splitting up the responsibilities of healthcare among PHC team members^11^. Everyone is expected to take part and collaborate with the planning and development of actions of dental health that are delivered to the population. The interdisciplinary approach in practice is favored by the valorization of multi-professional work which favors comprehensiveness and, consequently, the quality of care^2^. The results presented here confirm this assumption, as management variables were associated with better outcomes. For example, availability of information for the analysis of health situation and monitoring of dental health indicators was relevant for the provision of curative actions.

The comprehensiveness of actions in primary care depends on the qualification of the dentist, either through training or permanent education. This qualification aims to stimulate teamwork to foster dialogue among health professionals. Permanent education places the daily work as an object of reflection and evaluation. It is developed from the problems faced in the daily life and proposes that the processes of education of the workers are made from the problematization of the work process and that their needs of qualification and development are defined by the health needs of the population^8^.

The main limitation of this study is that it analyzed DHT that voluntarily adhered to PMAQ-AB. On the other hand, the study has strong points. The sample size provides power to the study, as 81.5% of the DHT of Brazil were assessed. Due to the national scope of the study, information bias may have occurred, since different teams of evaluators with a uniform training lead the evaluation. However, the bias may have been minimized by the size of the sample and by the fact that all equipment, instruments, and supplies were verified *in loco*.

In conclusion, the majority of the PHC services did not present the minimum equipment to provide the curative actions evaluated while only 14.8% performed them considering the presence of a minimum set of materials. Better results were found in DHT that had oral health technician, with higher workload and that plan their activities, as well as in those that had dentists with better working contracts, with degrees in public health and with permanent education.
